# Supplementary material for: Epigenome-wide association studies identify DNA methylation associated with kidney function
Source: Nat Commun. 2017 Nov 3;8:1286. doi: 10.1038/s41467-017-01297-7 (PMC5668367; doi:10.1038/s41467-017-01297-7)
Supplement: Supplementary file 1 — Supplementary Information [file 41467_2017_1297_MOESM1_ESM.pdf]

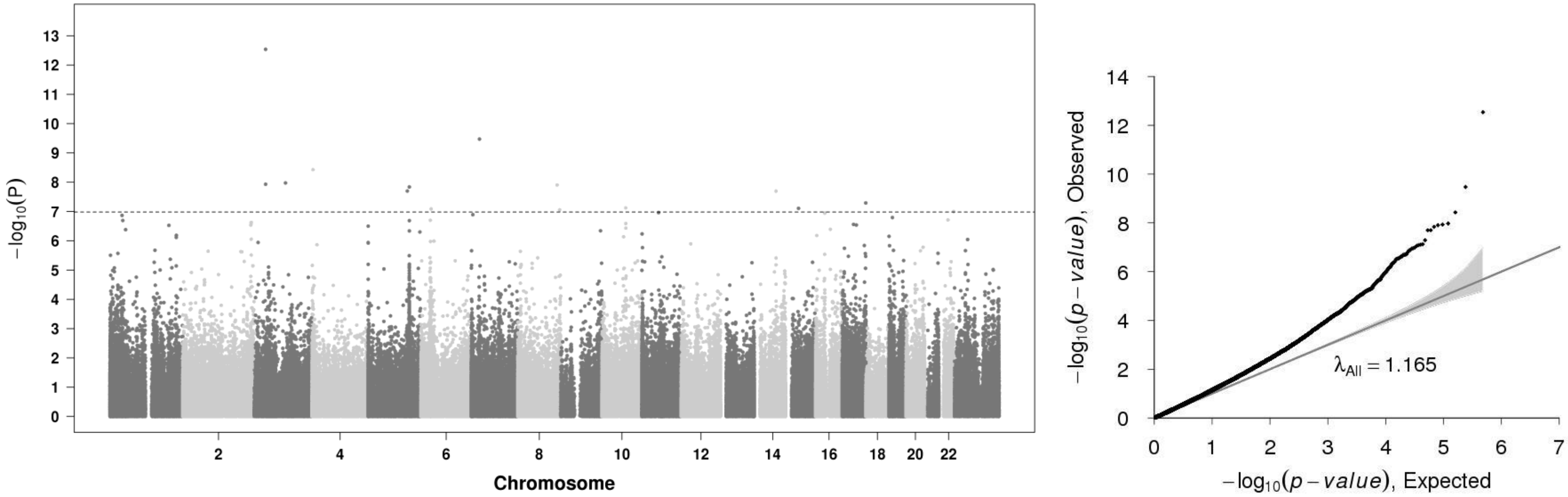

Supplementary Figure 1. Manhattan plot and QQ plot from EWAS of eGFR among 2,264 African American participants in the ARIC study.

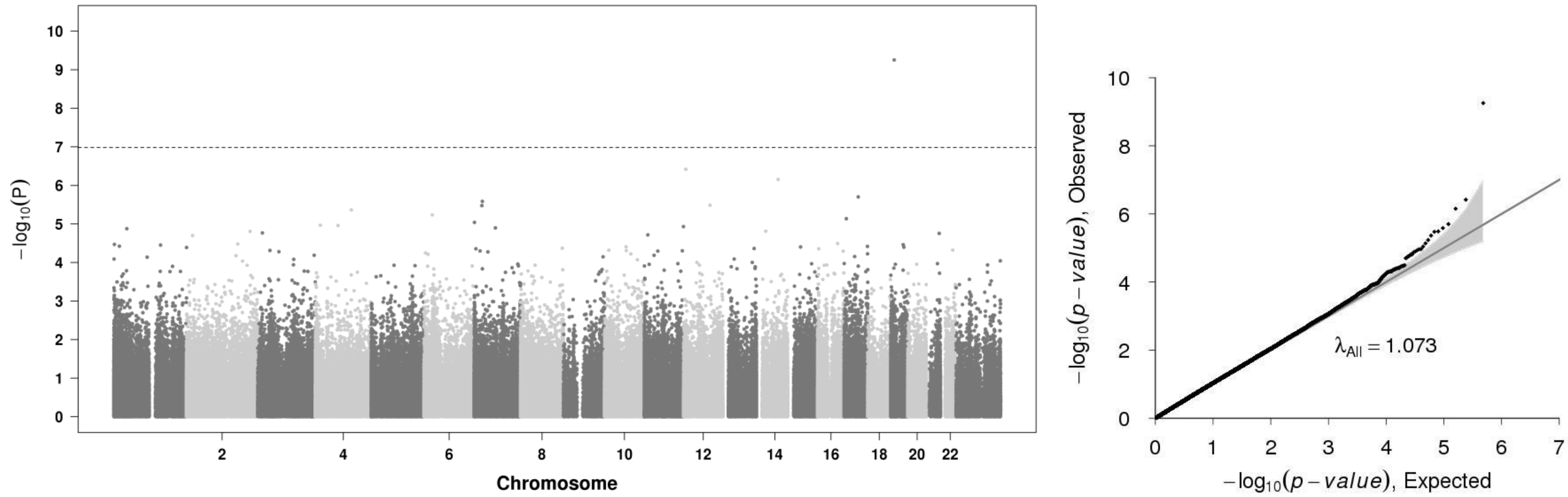

Supplementary Figure 2. Manhattan plot and QQ plot from EWAS of prevalent CKD among 2,264 African American participants in the ARIC study ($N_{CKDcases}$=75).

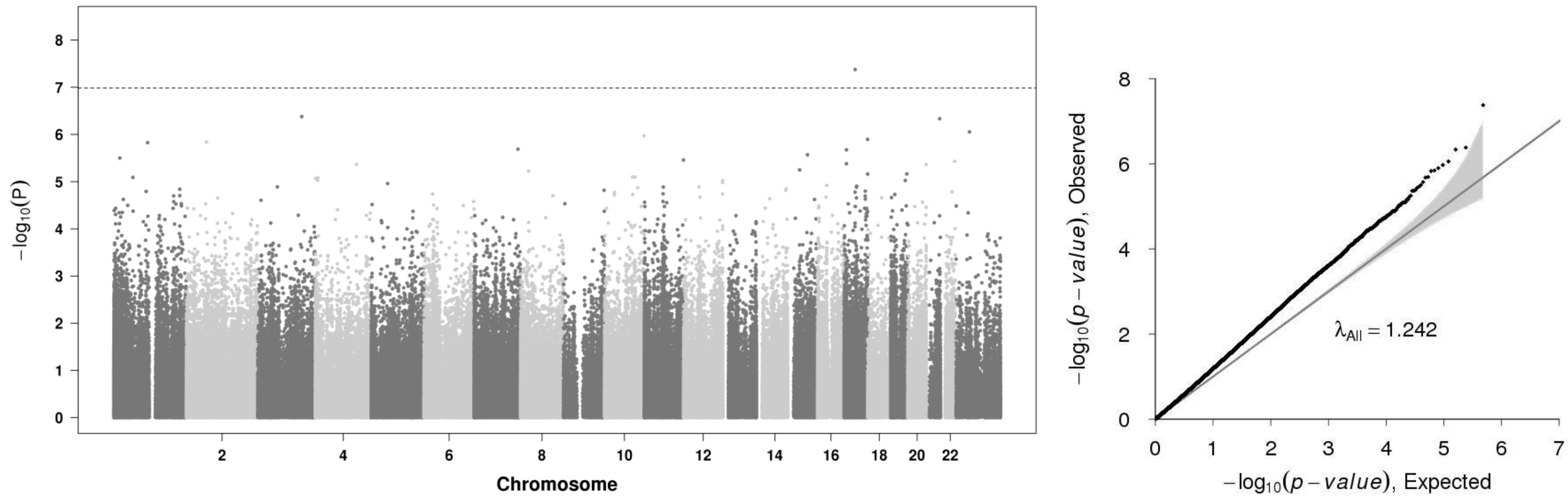

Supplementary Figure 3. Manhattan plot and QQ plot from EWAS of incident CKD among 2,189 African American participants free of CKD at baseline in the ARIC study ($N_{CKDicases}$=301).

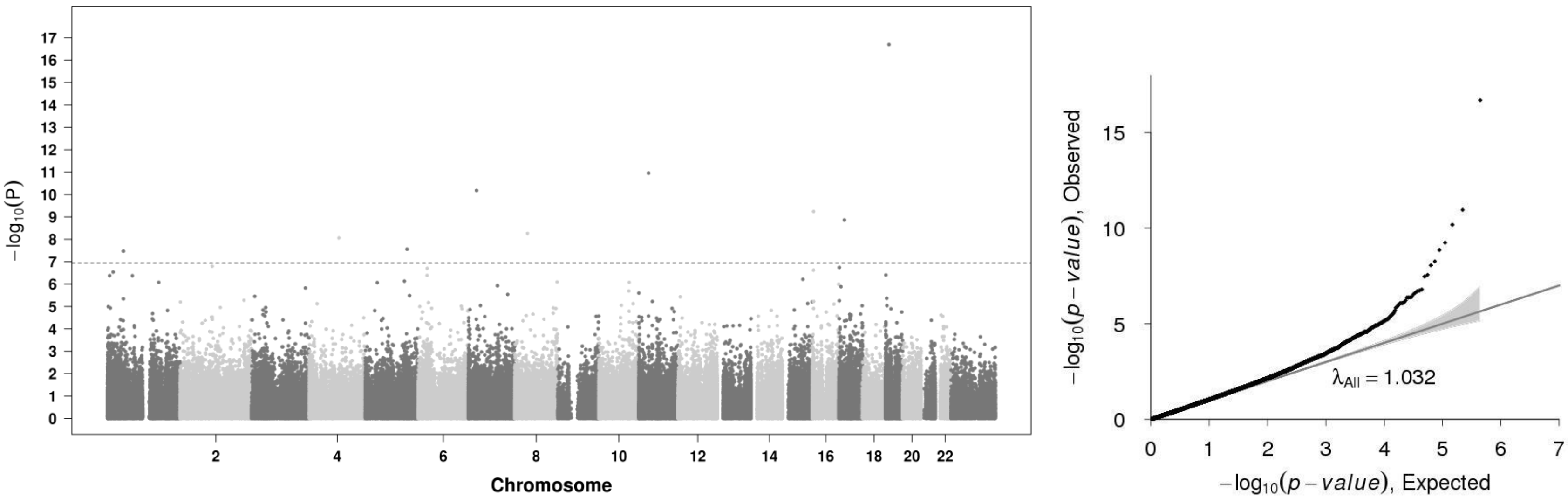

Supplementary Figure 4. Manhattan plot and QQ plot from EWAS of eGFR among 2,595 European ancestry participants in the Framingham Heart Study.

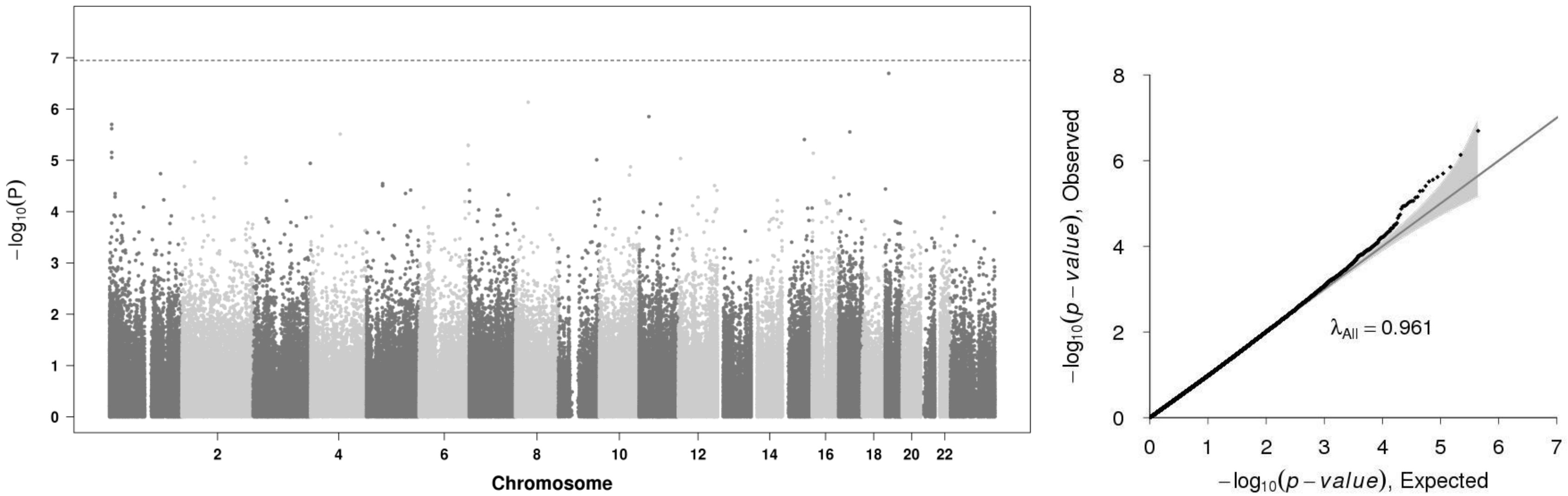

Supplementary Figure 5. Manhattan plot and QQ plot from EWAS of prevalent CKD among 2,595 European ancestry participants in the Framingham Heart Study ($N_{CKDcases}$=298).

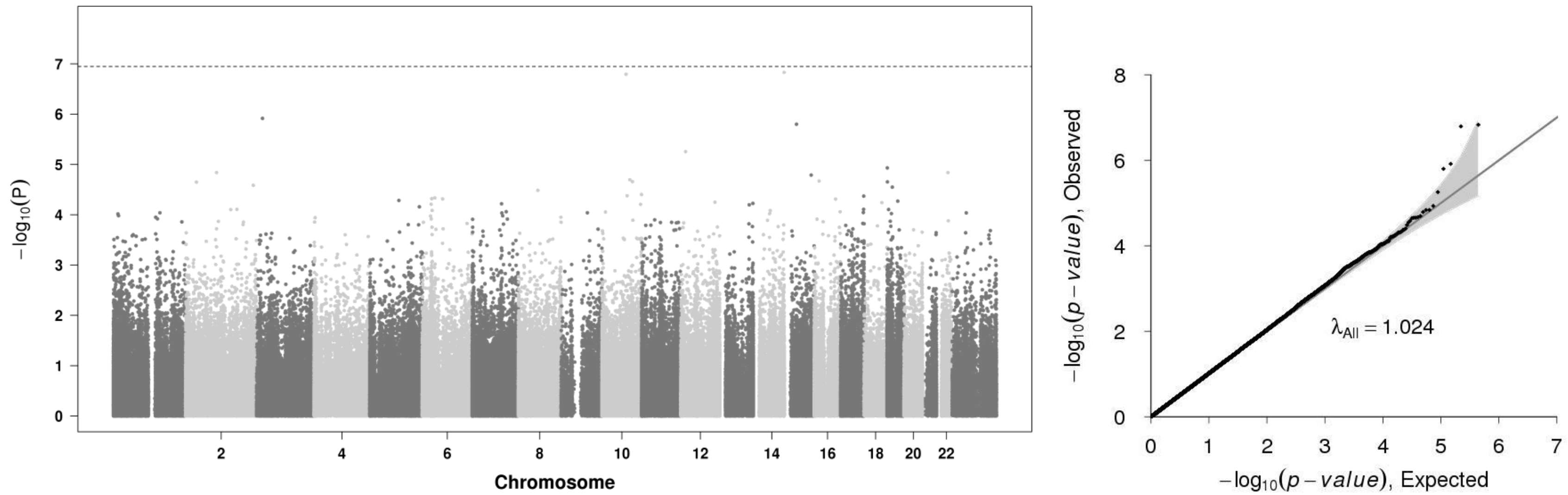

Supplementary Figure 6. Manhattan plot and QQ plot from EWAS of incident CKD among 1,386 European ancestry participants free of CKD at baseline in the Framingham Heart Study ($N_{CKDicases}$=232).

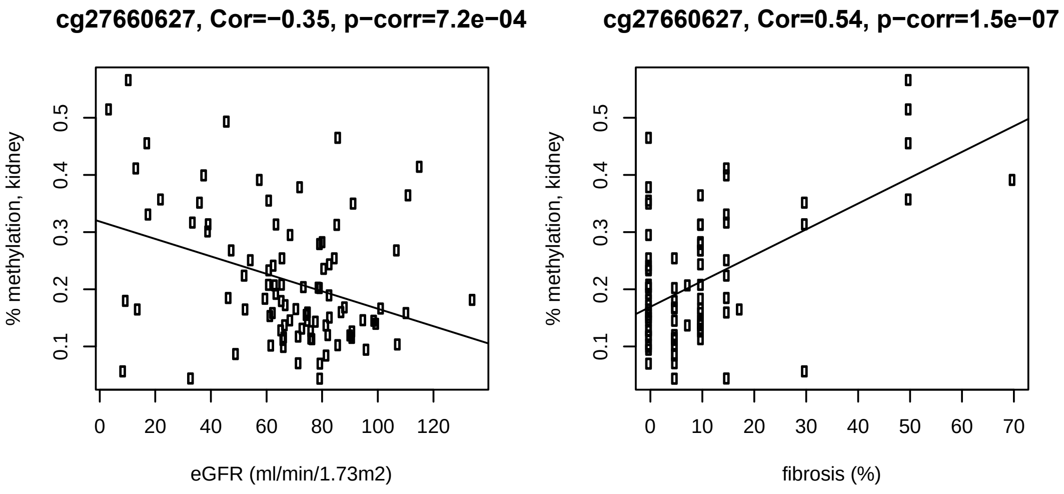

Supplementary Figure 7. DNA methylation quantified from kidney tissue and correlation with eGFR and % fibrosis for cg27660627.

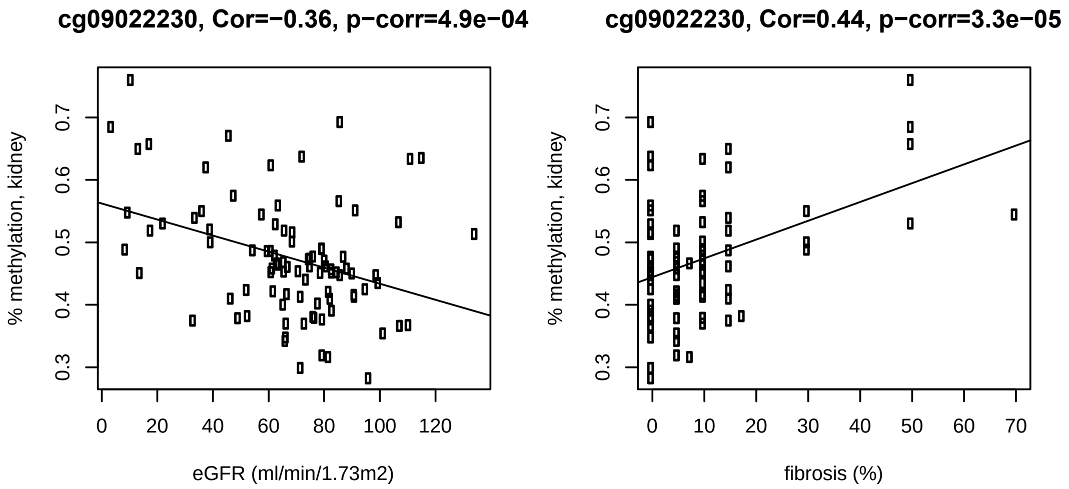

Supplementary Figure 8. DNA methylation quantified from kidney tissue and correlation with eGFR and % fibrosis for cg09022230.

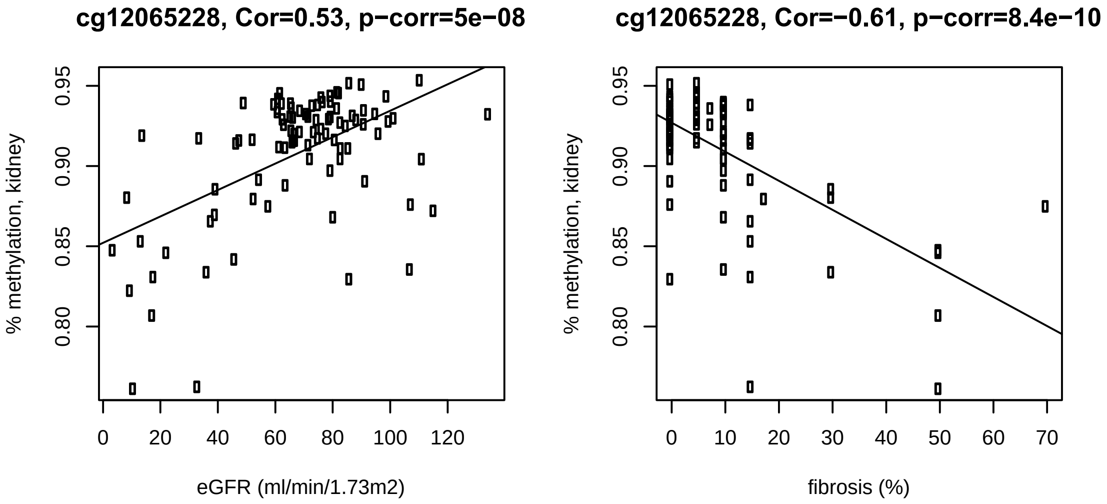

Supplementary Figure 9. DNA methylation quantified from kidney tissue and correlation with eGFR and % fibrosis for cg12065228.

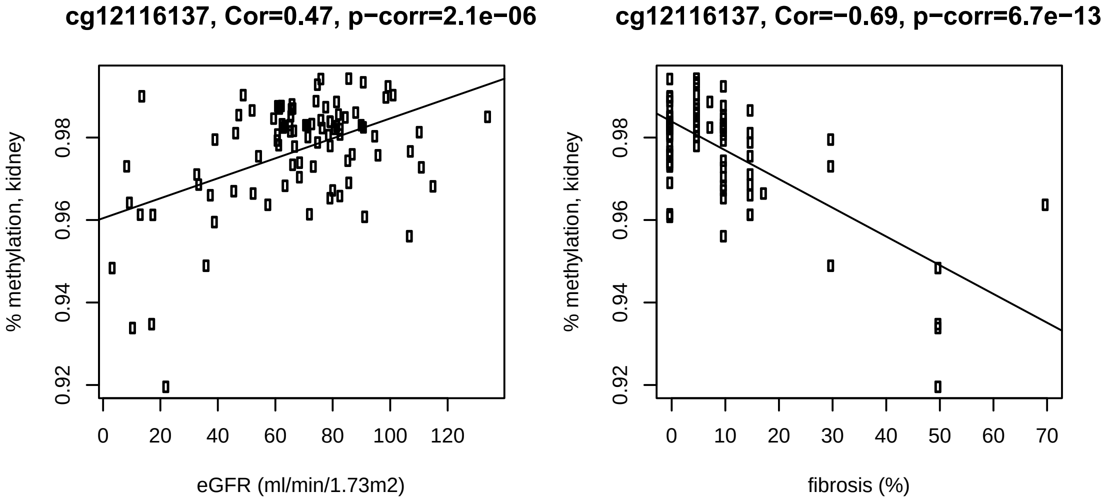

Supplementary Figure 10. DNA methylation quantified from kidney tissue and correlation with eGFR and % fibrosis for cg12116137.

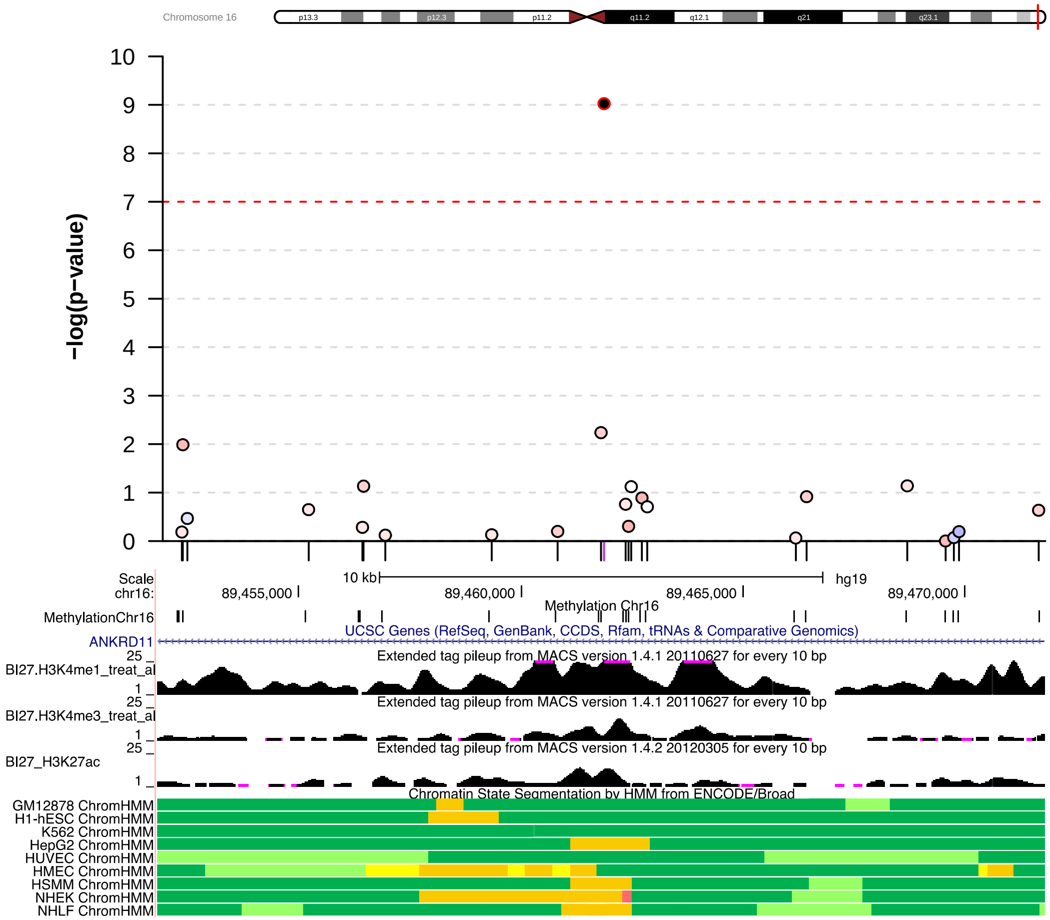

Supplementary Figure 11. Regional association plots for cg27660627 at the ANKRD11 locus and surrounding CpGs and annotation by chromatin annotation from various tissues and histone modifications from human kidney cortex. H3K4me1 marks represent poised enhancer elements, H3K4me3 marks represent transcription start sites for actively transcribed genes, and H3K27ac marks represent active enhancer elements. Chromatin annotation track color-code: dark red: active promoter, light red: weak promoter, dark green: transcriptional transition/elongation, light green: weak transcribed, yellow: weak/poised enhancer, orange: strong enhancer, blue: insulator, grey: repressed polycomb, light grey: heterochromatin;low signal;repetitive/CNV. Chromatin annotation tracks were evaluated from 9 different cell lines from the ENCODE Project: B-lymphoblastoid cells (GM12878), embryonic stem cells (H1 hESC), erythrocytic leukemia cells (K562), hepatocellular carcinoma cells (HepG2), umbilical vein endothelial cells (HUVEC), mammary epithelial cells (HMEC), skeletal muscle myoblasts (HSMM), normal epidermal keratinocytes (NHEK), and normal lung fibroblasts (NHLF).

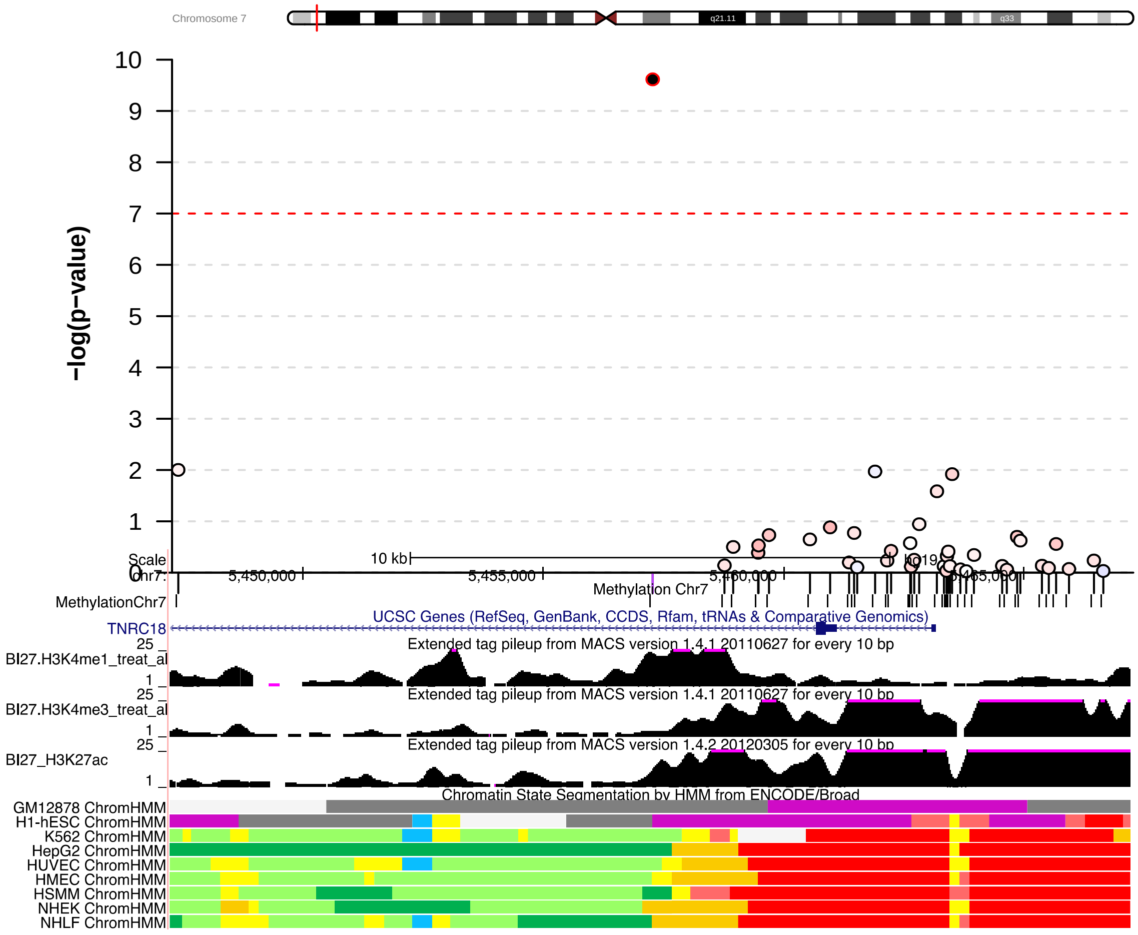

Supplementary Figure 12. Regional association plots for cg09022230 at the TNRC18 locus and surrounding CpGs and annotation by chromatin annotation from various tissues and histone modifications from human kidney cortex. H3K4me1 marks represent poised enhancer elements, H3K4me3 marks represent transcription start sites for actively transcribed genes, and H3K27ac marks represent active enhancer elements. Chromatin annotation track color-code: dark red: active promoter, light red: weak promoter, dark green: transcriptional transition/elongation, light green: weak transcribed, yellow: weak/poised enhancer, orange: strong enhancer, blue: insulator, purple: inactive/poised promoter, grey: repressed polycomb, light grey: heterochromatin;low signal;repetitive/CNV. Chromatin annotation tracks were evaluated from 9 different cell lines from the ENCODE Project: B-lymphoblastoid cells (GM12878), embryonic stem cells (H1 hESC), erythrocytic leukemia cells (K562), hepatocellular carcinoma cells (HepG2), umbilical vein endothelial cells (HUVEC), mammary epithelial cells (HMEC), skeletal muscle myoblasts (HSMM), normal epidermal keratinocytes (NHEK), and normal lung fibroblasts (NHLF).

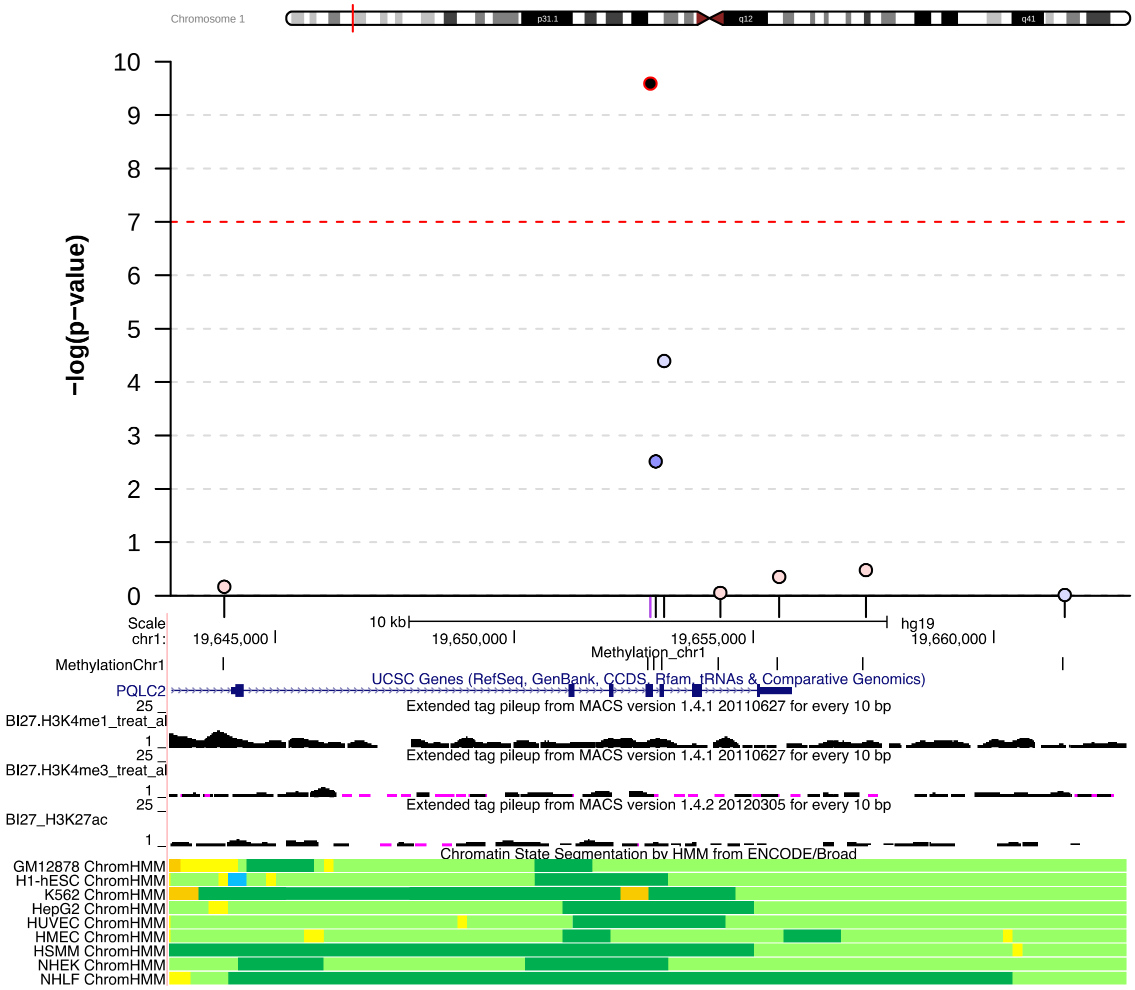

Supplementary Figure 13. Regional association plots for cg12065228 at the PQLC2 locus and surrounding CpGs and annotation by chromatin annotation from various tissues and histone modifications from human kidney cortex. H3K4me1 marks represent poised enhancer elements, H3K4me3 marks represent transcription start sites for actively transcribed genes, and H3K27ac marks represent active enhancer elements. Chromatin annotation track color-code: dark red: active promoter, light red: weak promoter, dark green: transcriptional transition/elongation, light green: weak transcribed, yellow: weak/poised enhancer, orange: strong enhancer, blue: insulator, grey: repressed polycomb, light grey: heterochromatin;low signal;repetitive/CNV. Chromatin annotation tracks were evaluated from 9 different cell lines from the ENCODE Project: B-lymphoblastoid cells (GM12878), embryonic stem cells (H1 hESC), erythrocytic leukemia cells (K562), hepatocellular carcinoma cells (HepG2), umbilical vein endothelial cells (HUVEC), mammary epithelial cells (HMEC), skeletal muscle myoblasts (HSMM), normal epidermal keratinocytes (NHEK), and normal lung fibroblasts (NHLF).

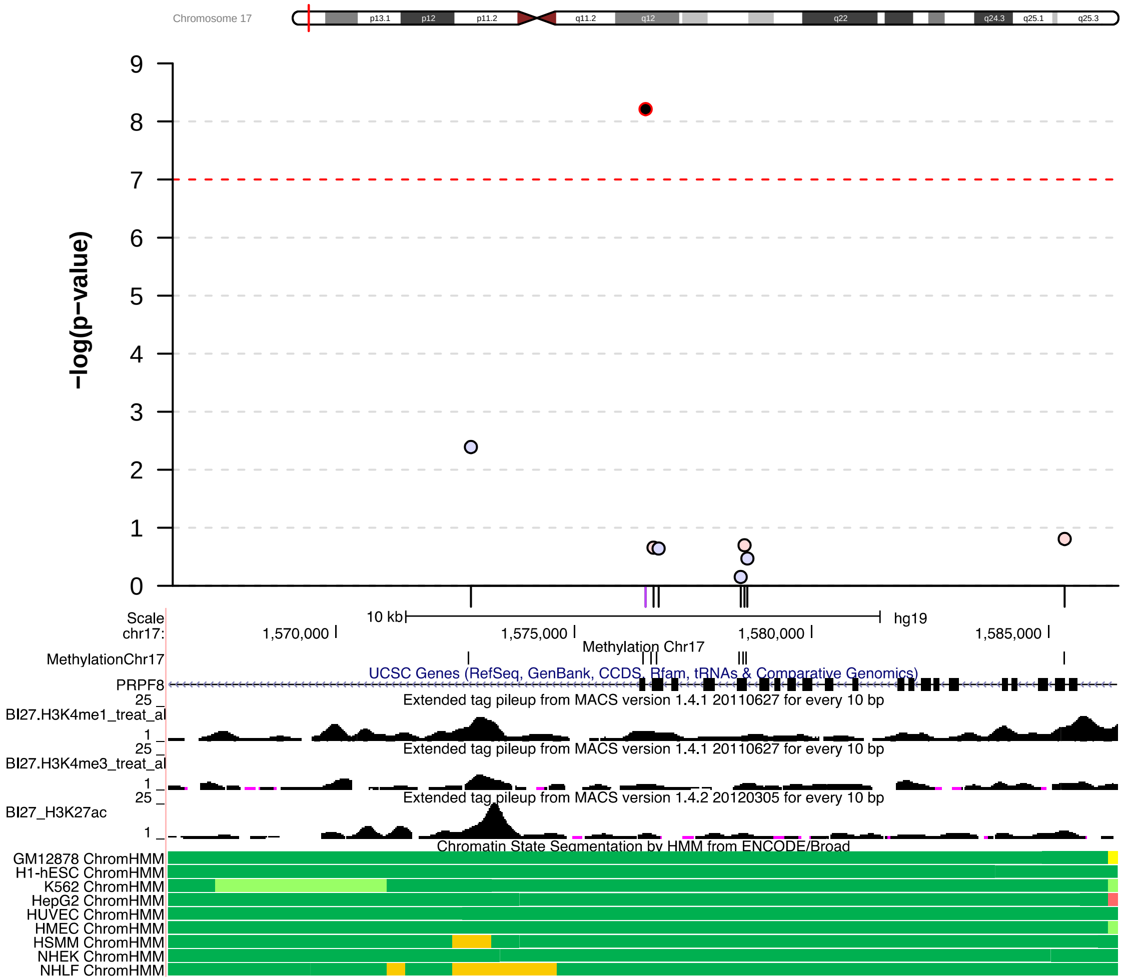

Supplementary Figure 14. Regional association plots for cg12116137 at the PRPF8 locus and surrounding CpGs and annotation by chromatin annotation from various tissues and histone modifications from human kidney cortex. H3K4me1 marks represent poised enhancer elements, H3K4me3 marks represent transcription start sites for actively transcribed genes, and H3K27ac marks represent active enhancer elements. Chromatin annotation track color-code: dark red: active promoter, light red: weak promoter, dark green: transcriptional transition/elongation, light green: weak transcribed, yellow: weak/poised enhancer, orange: strong enhancer, blue: insulator, grey: repressed polycomb, light grey: heterochromatin;low signal;repetitive/CNV. Chromatin annotation tracks were evaluated from 9 different cell lines from the ENCODE Project: B-lymphoblastoid cells (GM12878), embryonic stem cells (H1 hESC), erythrocytic leukemia cells (K562), hepatocellular carcinoma cells (HepG2), umbilical vein endothelial cells (HUVEC), mammary epithelial cells (HMEC), skeletal muscle myoblasts (HSMM), normal epidermal keratinocytes (NHEK), and normal lung fibroblasts (NHLF).

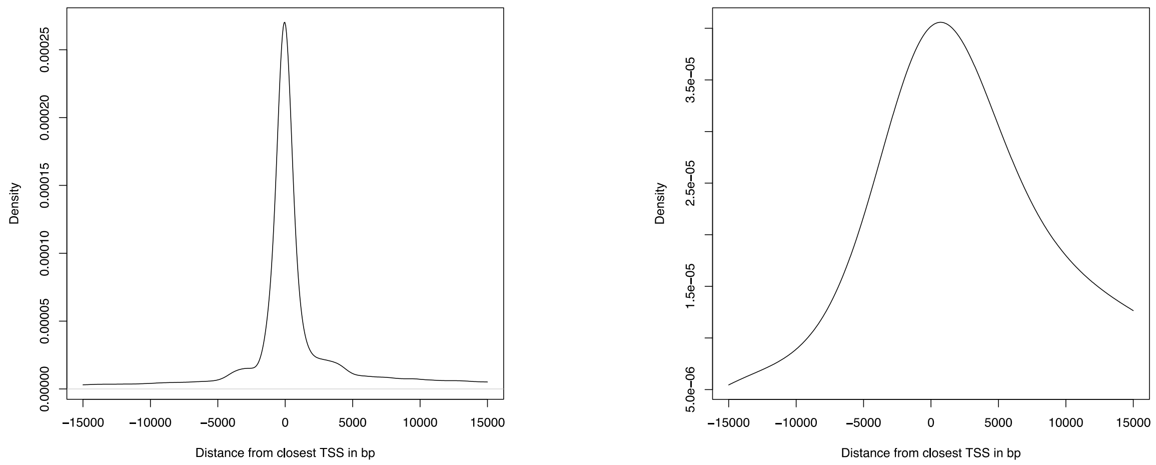

	Other	CpG Island	North shore	South shore	North shelf	South shelf	Pro-moter	Gene body	Inter-genic
Odds Ratio	**1.72**	**0.21**	1.30	0.70	**2.57**	1.51	**0.29**	**2.68**	0.79
P-value	**0**	**0**	0.1	0.1	**0**	0.1	**0**	**0**	0.1

Supplementary Figure 15. Distribution of distance to the nearest transcription start site for all 430,169 CpGs in the meta-analysis (left side) and for the 243 eGFR-associated CpGs at p<1e-05 (right side). Below a table of odds ratios and empirical p-values (enrichment or depletion), comparing eGFR-associated sites to background, for genomic localization with respect to CpG island and gene region annotation categories.
