# Supplementary material for: Epigenome-wide association studies identify DNA methylation associated with kidney function
Source: Nat Commun. 2017 Nov 3;8:1286. doi: 10.1038/s41467-017-01297-7 (PMC5668367; doi:10.1038/s41467-017-01297-7)
Supplement: Supplementary file 3 — Description of Additional Supplementary Files [file 41467_2017_1297_MOESM3_ESM.pdf]

Description of Additional Supplementary Files

File Name: Supplementary Data 1
Description: DNA Methylation sites associated with eGFR at p<1e-5 in the ARIC study with replication results in the FHS.

File Name: Supplementary Data 2
Description: DNA Methylation sites associated with prevalent CKD at p<1e-5 in the ARIC study with replication results in the FHS.

File Name: Supplementary Data 3
Description: DNA Methylation sites associated with incident CKD at p<1e-5 in the ARIC study with replication results in the FHS.

File Name: Supplementary Data 4
Description: DNA Methylation sites associated with eGFR at p<1e-5 in FHS with replication results in ARIC.

File Name: Supplementary Data 5
Description: DNA Methylation sites associated with prevalent CKD at p<1e-5 in FHS with replication results in ARIC.

File Name: Supplementary Data 6
Description: DNA Methylation sites associated with incident CKD at p<1e-5 in FHS with replication results in ARIC.

File Name: Supplementary Data 7
Description: Probe characteristics of replicated DNA methylation sites in the ARIC study and FHS.

File Name: Supplementary Data 8
Description: Information on genes located near the validated eGFR- or CKD-associated CpGs.

File Name: Supplementary Data 9
Description: Sensitivity analysis of replicated CpGs associated with eGFR or prevalent CKD.

File Name: Supplementary Data 10
Description: Association between replicated DNA methylation sites and incident CKD in the ARIC study and FHS.

File Name: Supplementary Data 11
Description: Investigation of eGFR-SNP associations in the 1 MB regions surrounding the 19 validated CpGs.

File Name: Supplementary Data 12
Description: Association of previously reported CKD-associated CpGs and renal traits in ARIC and FHS.

File Name: Supplementary Data 13
Description: DNA Methylation sites associated with eGFR at p<1e-5 in the meta analysis.

File Name: Supplementary Data 14
Description: DNA Methylation sites associated with CKD at p<1e-5 in the meta analysis.

File Name: Supplementary Data 15
Description: DNA Methylation sites associated with iCKD at p<1e-5 in the meta analysis.

File Name: Supplementary Data 16
Description: eGFR-associated CpGs mapping into the binding sites for each of the three significantly enriched transcription factors.

File Name: Supplementary Data 17
Description: Pathways from KEGG associated with eGFR at a false discovery rate of <0.01.
